# Identification of Novel Tumor Markers in Prostate, Colon and Breast Cancer by Unbiased Methylation Profiling

**DOI:** 10.1371/journal.pone.0002079

**Published:** 2008-04-30

**Authors:** Woonbok Chung, Bernard Kwabi-Addo, Michael Ittmann, Jaroslav Jelinek, Lanlan Shen, Yinhua Yu, Jean-Pierre J. Issa

**Affiliations:** 1 Department of Leukemia, Baylor College of Medicine, Houston, Texas, United States of America; 2 Department of Pathology, Baylor College of Medicine, Houston, Texas, United States of America; 3 Department of Experimental Therapeutics, Monroe Dunaway (M. D.) Anderson Cancer Center, Baylor College of Medicine, Houston, Texas, United States of America; Centre National de la Recherche Scientifique, France

## Abstract

DNA hypermethylation is a common epigenetic abnormality in cancer and may serve as a useful marker to clone cancer-related genes as well as a marker of clinical disease activity. To identify CpG islands methylated in prostate cancer, we used methylated CpG island amplification (MCA) coupled with representational difference analysis (RDA) on prostate cancer cell lines. We isolated 34 clones that corresponded to promoter CpG islands, including 5 reported targets of hypermethylation in cancer. We confirmed the data for 17 CpG islands by COBRA and/or pyrosequencing. All 17 genes were methylated in at least 2 cell lines of a 21-cancer cell line panel containing prostate cancer, colon cancer, leukemia, and breast cancer. Based on methylation in primary tumors compared to normal adjacent tissues, NKX2-5, CLSTN1, SPOCK2, SLC16A12, DPYS and NSE1 are candidate biomarkers for prostate cancer (methylation range 50%–85%). The combination of NSE1 or SPOCK2 hypermethylation showed a sensitivity of 80% and specificity of 95% in differentiating cancer from normal. Similarly NKX2-5, SPOCK2, SLC16A12, DPYS and GALR2 are candidate biomarkers for colon cancer (methylation range 60%–95%) and GALR2 hypermethylation showed a sensitivity of 85% and specificity of 95%. Finally, SLC16A12, GALR2, TOX, SPOCK2, EGFR5 and DPYS are candidate biomarkers for breast cancer (methylation range 33%–79%) with the combination of EGFR5 or TOX hypermethylation showing a sensitivity of 92% and specificity of 92%. Expression analysis for eight genes that had the most hypermethylation confirmed the methylation associated silencing and reactivation with 5-aza-2′-deoxycytidine treatment. Our data identify new targets of transcriptional silencing in cancer, and provide new biomarkers that could be useful in screening for prostate cancer and other cancers.

## Introduction

Prostate cancer is one of the most common malignancies and the second leading cause of cancer death among men in the United States [1]. The molecular mechanisms of prostate cancer development and progression remain poorly understood. Genetic and epigenetic alterations contribute to prostate cancer formation, as with most other cancers. DNA hypermethylation is the most common epigenetic abnormality in cancer. Hypermethylation of CpG islands in promoter regions usually results in gene silencing, and several tumor suppressor genes are hypermethylated in their promoter regions in human cancers, which is thought to contribute to tumorigenesis [2], [3]. Therefore, DNA hypermethylation may serve as a useful target to clone novel tumor suppressor genes. In prostate cancers, inactivation by aberrant methylation has been reported for many genes, such as APC [4], HIC1 [5], RARβ2 [6], GSTP1 [7], CDH1 [8], MDR1 [9] and RASSF1A [10].

Although the list of aberrantly methylated genes is expanding, only a few genes show promise as tumor biomarkers for early diagnosis and risk assessment of prostate cancer. Thus, large-(genome wide) scale screening of aberrant methylation of CpG islands is needed to identify prostate-specific epigenetic markers. To identify CpG islands differentially methylated in prostate cancer and other cancers, we performed methylated CpG island amplification (MCA) coupled with representational difference analysis (RDA) [11]. Applying this method to prostate cancer, we isolated 34 clones that corresponded to promoter CpG islands. Several are promising biomarkers for prostate and other cancers.

## Materials and Methods

### Cell lines and tissue samples

Cell lines used in this study were obtained from the American Type Culture Collection (Manassas, VA). All cell lines were cultured in recommended medium in the presence of 10% FBS in a humidified atmosphere containing 5% CO_2_ at 37°C. Samples of colon cancer paired with normal colon mucosa and samples of breast cancer paired with normal breast were obtained from established tissue banks at the University of Texas M. D. Anderson Cancer Center (Houston, TX). Samples of prostate cancer and normal prostate were obtained from Baylor College of Medicine (Houston, TX). Microdissection was employed to isolate prostate cancer cells and non-neoplastic prostate epithelial cells. SVHUC, normal urothelial cells, immortalized in vitro by SV40 was a gift from Dr. Bogdan Czerniak (M. D. Anderson Cancer Center). DNA was isolated by extraction buffer with 100 µg/ml proteinase K (Sigma, St. Louis, MO) and standard phenol-chloroform methods. All samples were collected from consenting patients according to institutional guidelines.

### MCA/RDA

MCA/RDA was performed as described [11]. Briefly, 5 µg of DNA was digested with *Sma*I followed by *Xma*I (New England Biolabs, MA). The restriction fragments were ligated to adapters and amplified by PCR. The reaction mixture was incubated at 72°C for 5 min and at 95°C for 3 min, and then was subjected to 25 cycles of 1 min at 95°C and 3 min at 77°C followed by a final extension of 10 min at 77°C. MCA amplicons from the prostate cancer cell lines DU145, PC3 and LNCaP were mixed and used as the tester for RDA. MCA amplicons were generated from a mixture of normal blood and normal colon mucosa DNA was used as the driver. Two rounds of RDA subtractive hybridization were performed on these MCA amplicons using different adapters, JMCA and NMCA. After the third round of subtractive hybridization and selective amplification, the RDA products were cloned into a TOPO TA cloning vector (Invitrogen, Carlsbad, CA). Sequence analysis was carried out with an ABI PRISM 377 DNA sequencer (Applied Biosystems, Foster City, CA) at the DNA sequencing core facility at the University of Texas M. D. Anderson Cancer Center.

### DNA bisulfite treatment

After DNA extraction, DNA was treated with bisulfite as reported previously [12]. Two µg of genomic DNA were denatured by 0.2 M NaOH at 37°C for 10 minutes, followed by incubation with freshly prepared 30 µl 10 mM hydroquinone (Sigma) and 520 µl 3 M sodium bisulfite (pH 5.0) at 50°C for 16 hours. Bisulfite-converted DNA was purified with a Wizard miniprep Column (Promega) and incubated with 0.3 M NaOH for 5 minutes at room temperature. DNA was then precipitated with ammonium acetate and ethanol, washed with 70% ethanol, dried and dissolved in 40 µl distilled water. We used *Sss*I methylase (New England Biolabs) - treated normal leukocytes DNA as a positive control for methylation studies.

### Combined bisulfite restriction analysis (COBRA) and Pyrosequencing

We used bisulfite-PCR followed by COBRA [13] and/or Pyrosequencing [14] to analyze the methylation status of cell lines and patient samples. PCR reactions were carried in 50 µl reaction volume; including 2 µl bisulfite treated DNA, 2 mM MgCl_2_, 0.25 mM dNTP, 1 unit *Taq* polymerase, 16 mM (NH_4_)_2_SO_4_, 67 mM Tris-HCl (pH 8.8), 1 mM 2-mercaptoethanol and 100 nM primers. COBRA primers and restriction enzymes for digestion are shown in Table 1. PCR products after restriction enzyme digestion were separated in 5% nondenaturing polyacrylamide gels, followed by densitometric analysis to obtain quantitative methylation levels. Densitometric analysis was performed using a BioRad Geldoc 2000 digital analyzer equipped with the Quantity One version 4.0.3 software (BioRad, Hercules, CA). SLC16A12, TOX, GALR2, TFAP2C and PAX9 were analyzed by COBRA and/or quantitated using the PSQ HS 96 Pyrosequencing System (Pyrosequencing Inc, Westborough, MA) [14]. In order to determine the quantitative accuracy of the COBRA and pyrosequencing assays, we tested DNA from normal leukocytes spiked with 0%, 33%, 67% and 100% methylated DNA obtained by *Sss*I methylase treatment.

**Table 1:** Primers used in this study.

COBRA				
	Sence	Antisense	PCR size	Restriction Enzyme
NSE1	GAATTAAGGTTTTGGGGGATT	CCCAAAACACCCCAATATAA	109 bp	*HpyCH*4IV
TFAP2C	GGGGTGGGTTTTAGTATTTGG	CCAACCAATCCCAACCTAAC	224 bp	*Hpa*I
TFAP2E	GGGTTTATGGAGGTAGGA	AAAAACTACCATAACTTATCTCTTTA	179 bp	*Hpa*I
CDEP	TTTTGTTTGGGGTTGTTT	ACTCCCCCAAACTTAAATC	142 bp	*Hpy188*I
NKX2-5	GAGAGTAGGGTTGGGGAATATG	AACCCCTAACCCAATAACAAACT	236 bp	*Bst*UI
BTBD14A	GGGAGAGTTTTYGGGTTTTT	AAACCCAATCCAAACAACTTACA	238 bp	*Rsa*I
IRX5	GAGTAATTTTTGAGTTTAAGGTTTGG	AACAACCAAAAACCAAATACCC	527 bp	*HpyCH4*III
ETNK2	GAAGGGGGTATAGTTATTTTTAGTAG	TAAATCATAAACTTCCCCTAACC	126 bp	*Hinf*I
GALR2	GGTTAGGAGGAGGAGTAAGAGA	CTACACCCCTACCAAACTACAA	313 bp	*HpyCH*4IV
DPYS	TAGAATATTTGGGGTTTGAGTGT	TTAAAATACCCTCCTACAAAATCC	200 bp	*Hinf*I
CLSTN1	AAATTGGGAGGATTTTAAGATTT	CCCAAAACCCTTATCACTTC	385 bp	*HpyCH*4IV
SPOCK2	GGGGTTTTGATTTTTGTAGTATTTT	TTTCAAACAAATACATCTCCTAACC	165 bp	*Rsa*I
EGFR5	GAGTTGTTTTTAATTTGGATTTGTT	CAACTATATTCCTAACCCCAAAA	121 bp	*HpyCH*4IV
FOXN4	GGGTTTTTATTTTGGAAATGTA	CCCTACCCTATAACTAACCCTTA	291 bp	*Bst*BI
TOX1	GTGGTTTGTTTAAGAAGAAGAGGA	ACAAAACAACTCAAAATCTCCAAT	289 bp	*Taq*I
SLC16A12	GGGGTATGGGGGTGGTTT	ACCCAACCCAAACAAAACAAAT	397 bp	*Hpy188*I
PAX9	GGAAAGTTTTTGTTTGGGAGTG	AATAACATCAACAACCACCCAAT	284 bp	*Hinf*I

Pyro-sequencinig			
	Sense	Antisence	Sequencing primer
PAX9	TTTAGGTGGGGAGTTAGTTTGAAAGA	U-ACCAATCCCAAAACAAACTACATAATAATTAA	AAGGAGTTTTTTGGATTG
SLC16A12	TAGAGGGAGAGGTGGTTTAGGTGAT	U-CACCCAAATTAAAATCCCAAACTC	AAGGGTATTTTTTAAGGAAG
TOX	GGGGATAATGAGAGTATGAAGTTATTTGT	U-CCCTCACATCCCTTTATAATTTATTTAA	GGAGGAGGTGGAGTAGG
DPYS	TTAGTTTTTTAGGGGGGAGGAGT	U-ACCCCCCAACTCTACCTCAAAC	GTAGTATATGAGGTTAGGTTATAAAT
NKX2-5	AGAGTAGGGTTGGGGAATATGG	U-CCCTCTCCTACCCCTTATACTCAA	TAGGTGGGAGGTAGAA
SPOCK2	TAGAGGGAGGAGAGTTGAGGATAG	U-TCCACCTAAAAAAATCTTAACTTCTACAATA	GGAGGAGAGTTGAGGATAG
EGFR5	GGTTGGGGAAGTTAGTTGTAGAGG	U-ACCCAAAACTACTCCCAACTTAAATC	GGGAAGTTAGTTGTAGAGG
GALR2	GGGGTTAGGAGGAGGAGTAAGAGA	U-ACCACTCCCCAACCCTTCC	GGAGAGGTTGTTTTTAGTAG
TFAP2C	GGTTTTAGGGGAGGAGTTATGATAAT	U-CCCCCCAACAATATATCCTAA	TTATGATAATTTTTTTTTTATTAAG

RT-PCR			
	Sense	Antisence	PCR size
GALR2	GCACTTCCTCATCTTCCTCA	GACTGGCGGTAGTAGCTCAG	198 bp
DPYS	CTGTACGAAGCCTTCTCTCG	CCTTAGCTGCAGACTTGCTC	247 bp
TPAP2C	CCCACTGAGGTCTTCTGCTC	AGAGTCACATGAGCGGCTTT	245 bp
NSE1	CAGGAGACTTTAAAGGAGTTTGG	TCATCATCCGAGAAGAAGTAGG	206 bp
TFAP2E	GACCTGCAGGCAATGGAC	CCTTGTACTTGGACGTTGAGC	205 bp
CDEP	GGTACGATGATCAAGATGGAGA	CGTGGACTTTAAACTGATTGGA	223 bp
NKX2-5	CCCTAGAGCCGAAAAGAAAGA	GGTTCTGGAACCAGATCTTGAC	251 bp
SPOCK2	GAGACGAAGTGGAGGATGACTA	CTTGCAGATGGAGTCTTTGTTT	227 bp
GAPDH	ACAGTCAGCCGCATCTTCTT	GACAAGCTTCCCGTTCTCAG	259 bp

Y = C or T.

U = biotin labeled universal primer tag : 5′-biotin-GGGACACCGCTGATCGTTTA.

### RNA purification and reverse transcription-PCR (RT-PCR)

Gene expression was analyzed by RT-PCR. Total RNA obtained from different cell lines was isolated using the TRIZOL reagent (Molecular Research Center, Inc., Cincinnati, OH). Reverse transcription reactions were performed using MMLV-RT (Takara) and random hexamers on 5 µg of total RNA per reaction according to the manufacturer's protocol and amplified by PCR using primers shown in Table 1. GAPDH mRNA was used as an internal control.

### Treatment with *5-Aza-2′-deoxycytidine (5-AzadC)* and/or *Trichostatin A (TSA)*

Cancer cells were split 12–24 h before drug treatment. Cancer cells were treated with 5-AzadC (Sigma) and TSA (ICN, Irvine, CA) either alone or in combination. Cells were exposed continuously to 5-AzadC (1 µmol/L refreshed daily) for 3 days or to TSA (300 nmol/L) for 20 hours. For combined treatment, cells were cultured in the presence of 5-AzadC (1 µmol/L refreshed daily) for 3 days and exposed on the third day (24 hours) in combination with TSA (300 nmol/L). Mock-treated cells were cultured similarly.

### Statistical Analysis

Statistical analyses were performed using Prism 4 software (GraphPad Software, Inc., San Diego, CA). The Pearson test was used to determine correlations. The Fisher's exact test and t-tests were used to compare methylation in normal and cancer tissues. We used a cut-off corresponding to average methylation in normal tissues+2 standard deviation (*s_D_*) to call a cancer as methylation positive (above the cut-off). Sensitivity, specificity and accuracy of each individual or 2 combined candidate biomarkers were calculated. Sensitivity was defined as the number of true-positive (methylated in cancer) cases divided by the number of true-positive plus false-negative (not methylated in cancer) cases and specificity was defined as the number of true-negative (not methylated in normal tissue) cases divided by the number of true-negative plus false-positive (methylated in normal tissue) cases. The accuracy of a test is measured by the area under the Receiver Operating Characateristic (ROC) curve. All reported p values were 2-sided and *P*<0.05 was considered statistically significant.

## Results

### Detection of Methylated CpG Islands Using MCA-RDA

To identify novel CpG islands aberrantly methylated in prostate cancer, we used MCA-RDA [11]. RDA was performed on MCA amplicons from a mixture of the prostate cancer cell lines DU145, PC3 and LNCaP as a tester, and a mixture of normal colon mucosa DNA from three different men and normal leukocytes from two different men as a driver. After 2 rounds of RDA, PCR products were cloned and DNA sequencing was performed on 198 randomly selected clones. Of the 198 clones, 17 corresponded to repeats including *Alu*, 94 corresponded to non-CpG island DNA, 50 corresponded to non-promoter CpG islands and 37 corresponded to 34 unique promoter associated CpG islands, as revealed by BLAST (www.ncbi.nlm.nih.gov) and BLAT (genome.ucsc.edu) searches. The homology of each unique promoter associated clone, chromosomal location, and GenBank accession numbers are summarized in Table 2. Five of the 34 clones corresponded to genes previously reported as methylated in cancer. Four of the clones corresponded to bi-directional promoters. Chromosomal location of the clones appeared random with no evidence of clustering (Table 2).

**Table 2:** Hypermethylated promoter - associated CpG island clones isolated by MCA-RDA.

No.	Gene name	Bidirectional	Locus	Location*	Previously Reported [reference]
1	ALX3	No	1p13.3	110324472–110324835	Yes [29]
2	TFAP2E	No	1p34.3	35708094–35708319	No
3	CLSTN1	No	1p36.22	9818652–9818922	No
4	AW295421, BM809018	Yes	1q21.1	144745596–144745909	No
5	ETNK2	No	1q32.1	200852162–200852445	No
6	NSE1	No	2p24.3	14723003–14723236	No
7	AB029015	No	3p24.3	16900443–16900785	No
8	KIAA1729	No	4p16.1	10135303–10135580	No
9	BC048329	No	5q31.1	135556264–135556542	No
10	CDX1	No	5q32	149526986–149527183	Yes [30]
11	FLJ14166 (CCNJL)	No	5q33.3	159671339–159671601	No
12	NKX2-5 (CSX)	No	5q35.1	172593550–172593870	Yes [11]
13	AK128409	No	6p25.3	1565515–1565753	No
14	MOGAT3	No	7q22.1	100632085–100632363	No
15	TOX	No	8q12.1	60193205–60193432	No
16	DPYS	No	8q22.3	105548353–105548518	No
17	SCXA	No	8q24.3	145460987–145461257	No
18	BTBD14A	No	9q34.3	136211905–136212286	No
19	SPOCK2 (Testican-2)	No	10q22.1	73517716–73517874	No
20	SLC16A12	No	10q23.31	91285295–91285471	No
21	FOXN4	No	12q24.11	108209604–108209916	No
22	FLT1 (VEGFR-1), CR600638	Yes	13q12.3	27966278–27966465	Yes [31]
23	CDEP (FARP1 )	No	13q13.2	97592455–97592835	No
24	PAX9	No	14q13.3	36202075–36202382	No
25	EGFR5 (C14orf27)	No	14q21.1	37794235–37794429	No
26	IRX5, AF275804	Yes	16q12.2	53523634–53523826	No
27	GALR2	No	17q25.1	71581744–71581919	No
28	NFIC (AK129956)	No	19p13.3	3414086–3414270	No
29	LOC126147	No	19p13.33	53869474–53869704	No
30	BX537419, BQ068319	Yes	19q13.43	63550508–63550666	No
31	GCX1	No	20q13.12	41978149–41978397	No
32	TFAP2C	No	20q13.31	54634117–54634337	No
33	TIMP3	No	22q12.3	31522005–31522204	Yes [32]
34	PP2447	No	22q13.33	48925974–48926227	No

* Based on Blat (www.genome.ucsc.edu) search of the May 2004 assembly.

### COBRA and pyrosequencing analysis in Cell lines

**Figure 1:**
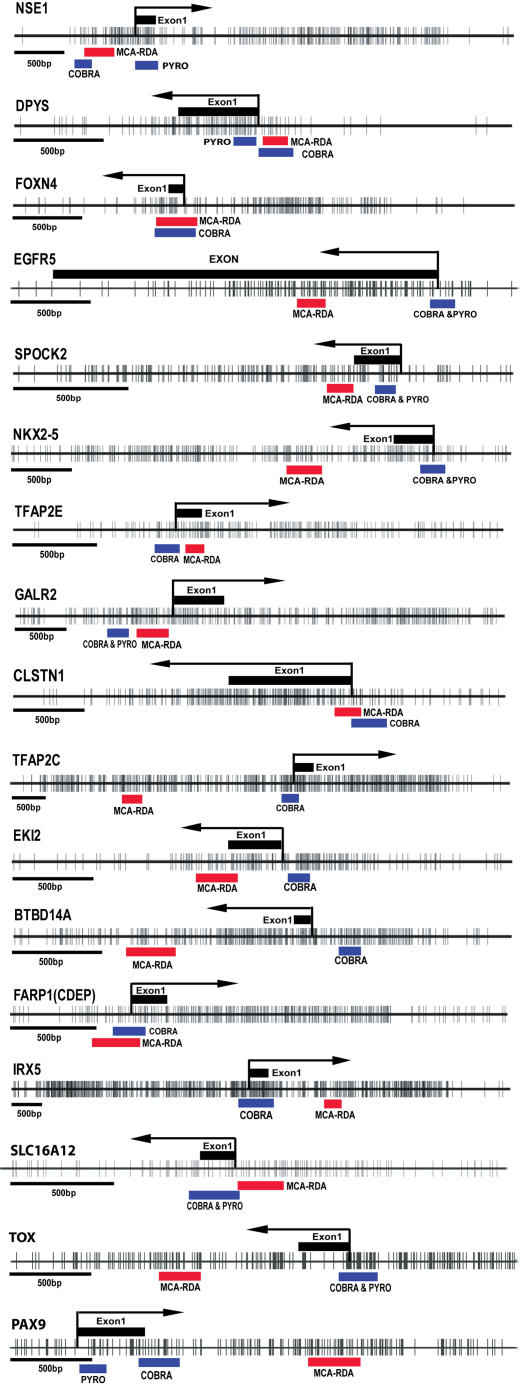
CpG island map and DNA methylation status of identified clones. CpG maps of the seventeen genes analyzed in this study. For each gene, short vertical bars indicate CpG sites. Exon1 is indicated by black rectangles at the top, whereas location of the MCA-RDA clones is indicated by red rectangles at the bottom. Arrows point to known or presumed transcription start sites. Blue bars at the bottom indicate areas that analyzed by COBRA or pyrosequencing.

**Figure 2:**
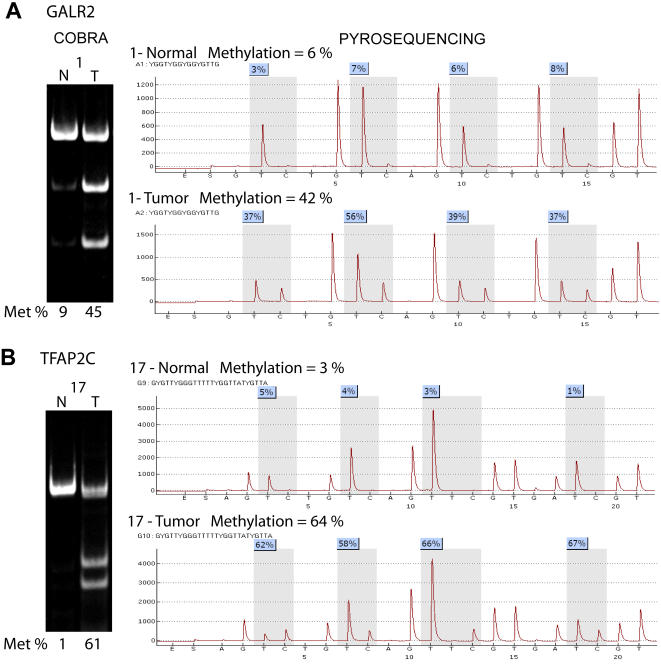
Representative COBRA and pyrosequencing for GALR2 and TFAP2C. For GALR2 and TFAP2C methylation analyses, we used COBRA (left panel) and pyrosequencing (right panel). The methylation density of pyrosequencing is presented in the top of each tracing as the averaged methylation of the CpG sites analyzed. Both methods analyzed almost the same sites in the promoter region as presented in Figure 1. A. In GALR2 methylation analysis, normal adjacent sample showed 9% methylation by COBRA and 6% by pyrosequencing but tumor sample showed 45% by COBRA and 42% by pyrosequencing. B. In TFAP2C methylation analysis, normal sample showed 1% methylation by COBRA and 3% methylation by pyrosequencing but tumor sample showed 61% by COBRA and 64% by pyrosequencing.

**Figure 3:**
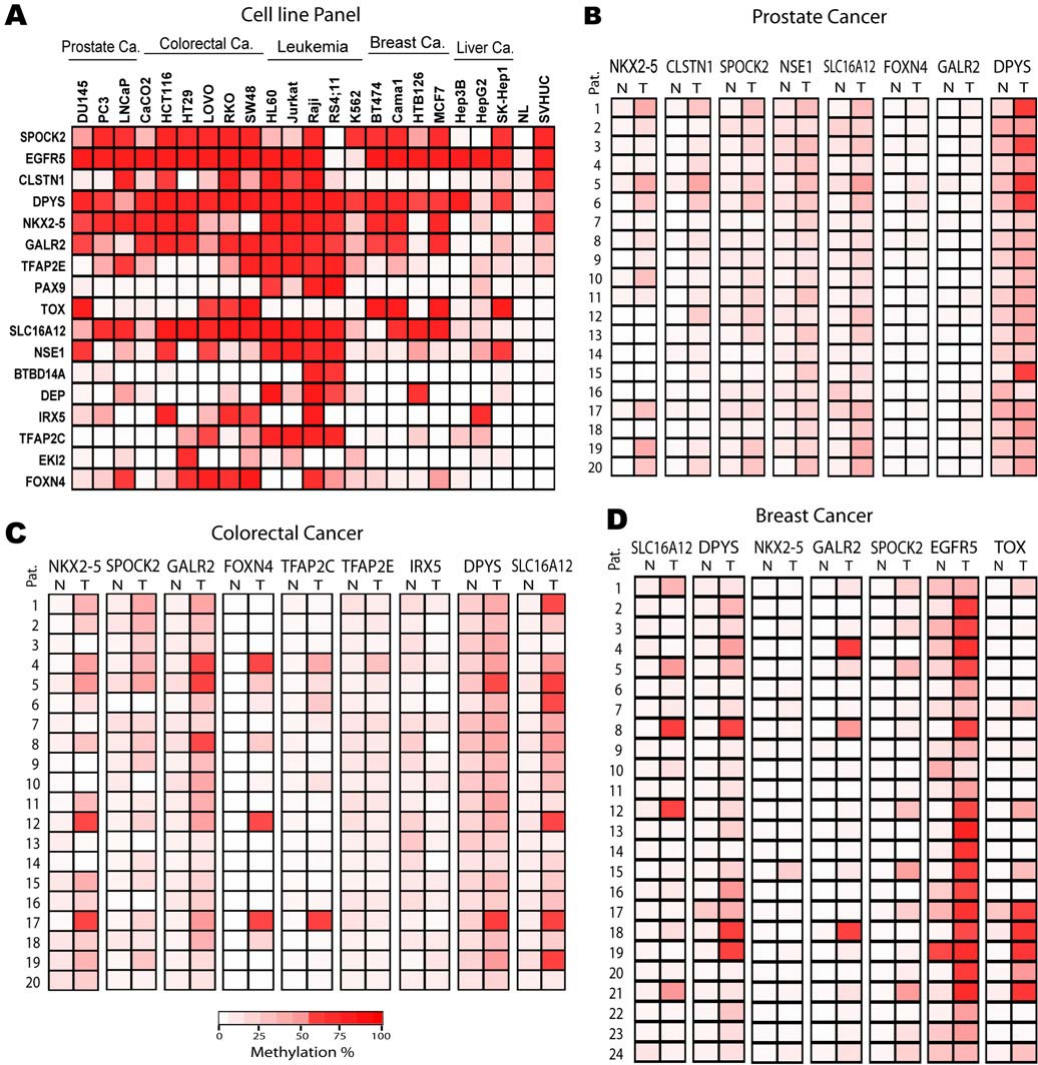
Heat map of DNA methylation profiling of identified clones by MCA-RDA. The bottom red-scale bar refers to the degree of methylation as measured by COBRA and/or pyrosequencing. NL represents normal leukocyte. A. All of the 17 genes investigated were methylated in at least 2 cell lines of a 21-cell line panel investigated. B. Methylation map for 8 genes studied in twenty primary prostate cancers (T) and paired normal (N) prostate samples. C. Methylation map for 9 genes studied in twenty primary colon cancers (T) and paired normal (N) colon samples. D. Methylation map of 7 genes in twenty-four breast cancers (T) and paired normal (N) samples. Pat.; patient samples.

To confirm the accuracy of MCA-RDA, differential methylation was investigated by COBRA analysis [15] or pyrosequencing for seventeen known genes (all except the 5 previouly known as methylated). All the MCA-RDA clones related to the genes analyzed in this study had CCCGGG sites on both sides consistent with the MCA technique. The primers for COBRA or pyrosequencing analysis were designed close to the promoter region in each case (Figure 1). Figure 2 shows examples of the COBRA and pyrosequencing analysis. The PCR product was digested with restriction enzymes that distinguish methylated from unmethylated DNA after bisulfite treatment for COBRA assay (Table 1). In order to determine the quantitative accuracy of the COBRA and pyrosequencing assays, we tested both assays on DNA from normal leukocytes, 2∶1 (33%) mixture, 1∶2 (67%) mixture and fully methylated DNA (100%) obtained by *Sss*I treatment. The assays showed significant correlations between predicted and measured values by pyrosequencing (GALR2, Pearson r  = 0.98, P<0.001) and COBRA (SLC16A12, Pearson r = 0.99, P = 0.008; GALR2, Pearson r = 0.99, P = 0.002). We also compared data obtained by COBRA or pyrosequencing in 364 measurements, and found a high degree of correlation between the two methods (Pearson r = 0.939, n = 364 pairs, P<0.001) (Figure 2). Similarly, correlations between two different pyrosequencing assays were very high (Pearson r = 0.977, n = 458 pairs, P<0.001). All of the 17 genes investigated were methylated in at least 2 cell lines of a 21-cell line panel (summarized in Figure 3A and Table S1). Sixteen of the 17 analyzed genes showed hypermethylation in at least one of three prostate cancer cell lines. Only TFAP2C did not display hypermethylation in any prostate cancer cell line. For this gene, the MCA product was 2 Kb upstream of the promoter and COBRA showed methylation close to the MCA product (data not shown) but not in the gene promoter. Individual cell lines varied in methylation frequency from 5/17 (Hep3B) to 15/17 (RAJI) and colon cancer and leukemia appeared to have the most methylation. Seven out of 17 genes analyzed in this study had methylation in the non-tumorigenic urothelial epithelium SVHUC cell line, which was immortalized by SV40. However, the AD12-SV40-immortalized PWR-1E normal human prostate cell line [16] did not show increased methylation of these genes (data not shown). Thus, acquired methylation in the SVHUC cell line could be derived from repeated passages and selection during cell culture.

### Analysis of Primary Prostate Cancer

**Figure 4:**
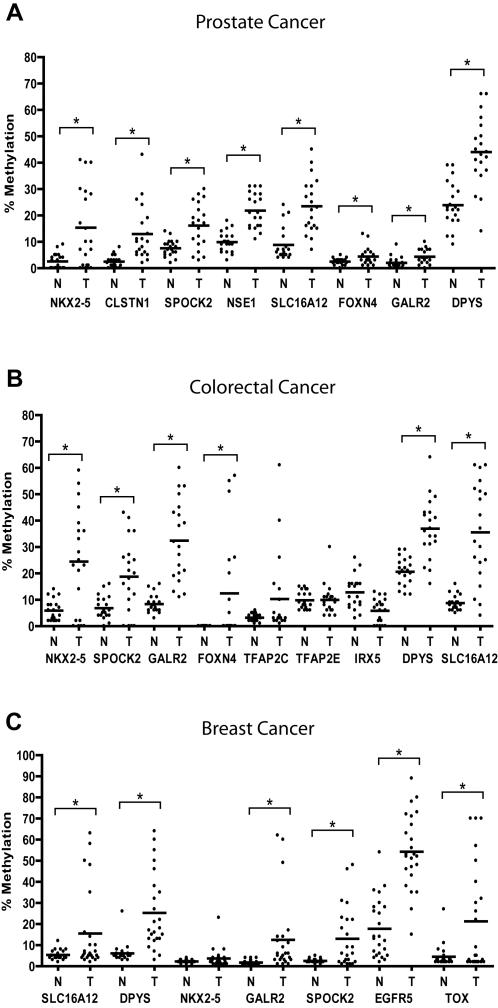
Scatter plots of methylation analysis in primary prostate cancer (A), colorectal cancer (B) and breast cancer (C). N and T represent tumor and adjacent normal. *Significantly different from normal adjacent samples, *P*<0.05.

Based on the CpG island methylation data from the 21-cell line panel, we selected 8 genes showing dense CpG island methylation in prostate cancer cell lines and further analyzed them in 20 primary prostate cancer and paired normal prostate samples. Compared to adjacent normal prostate, all 8 genes had significantly higher methylation in prostate cancer by t-test analysis (P<0.001 for NKX2-5; P<0.001 for SPOCK2; P = 0.004 for GALR2; P<0.001 for CLSTN1; P<0.001 for NSE1; P<0.001 for DPYS; P = 0.019 for FOXN4; P<0.001 for SLC16A12) (Figure 3B, 4A and Table S2). FOXN4 and GALR2 were relatively rarely methylated while the other 6 genes showed remarkably increased methylation levels. Of note, NSE1 and DPYS also showed some degree of methylation in normal prostate (average 10±4% for NSE1 and average 24±9% for DPYS). The combination of DPYS or NSE1 hypermethylation showed a sensitivity of 80%, specificity of 95% and accuracy of 95% and the combination of NSE1 or SPOCK2 hypermethylation showed a sensitivity of 80%, specificity of 95% and accuracy of 96% in differentiating prostate cancer from normal (Table 3).

**Table 3:** Diagnostic information of single or combined candidate biomarkers isolated by MCA-RDA.

	Biomarker	% Sensitivity	% Specificity	% Accuracy
***Prostate Ca.***				
	NKX2-5	55	95	79
	CALSTN1	65	95	92
	SPOCK2	60	95	80
	NSE1	65	95	95
	SLC16A12	55	95	90
	FOXN4	40	95	69
	GALR2	35	95	78
	DPYS	80	90	91
	DPYS or NKX2-5	85	95	90
	CALSTN1 or DYPS	75	95	94
	NSE1 or DPYS	80	95	95
	NSE1 or SPOCK2	80	95	96
	SLC16A12 or DPYS	75	95	93
***Colorectal Ca.***				
	NKX2-5	70	95	71
	SPOCK2	60	95	76
	GALR2	85	95	98
	FOXN4	35	100	70
	TFAP2C	30	95	58
	DPYS	80	90	91
	SLC16A12	85	95	91
	NKX2-5 or SLC16A12	90	95	92
	SPOCK2 or SLC16A12	90	95	94
	GALR2 or NKX2-5	90	95	96
	NKX2-5 or SPOCK2	80	95	89
***Breast Ca.***				
	SLC16A12	38	96	64
	DPYS	83	96	91
	TOX	42	96	63
	GALR2	67	88	87
	SPOCK2	63	96	73
	EGFR5	79	96	83
	DPYS or TOX	88	96	91
	A16 or SPOCK2	71	96	82
	A16 or DPYS	63	96	90
	EGFR5 or TOX	92	92	93

The other gene combinations did not improve the sensitivity markedly. The accuracy of a test is measured by the area under the Receiver Operating Characateristic (ROC) curve.

### Analysis of Methylation in Other Cancers

Next, we studied colon cancer and breast cancer. The DNA hypermethylation status of 9 genes showing dense CpG island methylation in colon cancer cell lines was further analyzed in 24 primary colon cancer and paired normal colon samples. In a panel of 20 colon cancer cases, all these genes except IRX5, TFAP2E and TFAP2C showed significantly higher methylation in cancer by t-test analysis (P<0.001 for NKX2-5, DPYS SPOCK2, GALR2 and SLC16A12; P = 0.008 for FOXN4) (Figure 3C, 4B and Table S3). Notably, GALR2 and DPYS showed some degree of methylation in normal colon (average 8±4% for GALR2 and average 21±5% for DPYS). GALR2 hypermethylation showed a sensitivity of 85%, specificity of 95% and accuracy of 98% and the combination of GALR2 or NKX2-5 hypermethylation showed a sensitivity of 90%, specificity of 95% and accuracy of 96% for detecting colorectal cancer (Table 3).

As shown in Figure 3D, the DNA hypermethylation status of 7 genes showing dense CpG island methylation in breast cancer cell lines was analyzed in 24 primary breast cancer and paired normal breast samples. Compared to adjacent normal breast, all 6 genes except NKX2-5 showed higher methylation in breast cancer by t-test analysis (P<0.001 for DPYS and EGFR5 P = 0.01 for SLC16A12; P = 0.002 for TOX; P = 0.003 for GALR2) (Figure 3B, 4C and Table S4). NKX2-5 was relatively rarely methylated while the other 6 genes showed remarkably increased methylation levels. Of note, EGFR5 showed some degree of methylation in normal breast (average 17±13%). The combination of EGFR5 or TOX hypermethylation showed a sensitivity of 92%, specificity of 92% and accuracy of 93% and the combination of DPYS or TOX hypermethylation showed a sensitivity of 88%, specificity of 96% and accuracy of 91% for detecting breast cancer (Table 3).

### Methylation and Gene Expression

**Figure 5:**
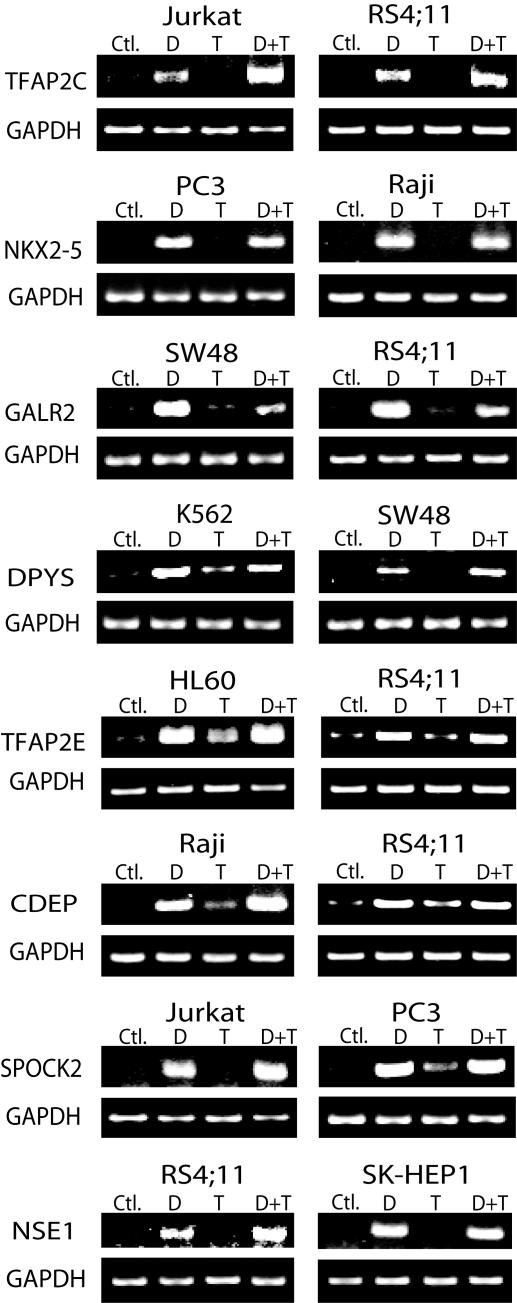
Silencing in cancer and reactivation of hypermethylation. Expression of target genes and GAPDH (control) was measured by RT-PCR in various cell lines. The sizes of the PCR products are listed in Table 1. Most cell lines chosen for analysis had high methylation level (>50%), and all had very low levels of expression at baseline, which was markedly increased by treatment with the methylation inhibitor, 5-azadC with or without the HDAC inhibitor TSA. Ctl.; no treatment, D; 5-AzadC alone, T; TSA alone, D+T; 5-AzadC and TSA in combination.

To confirm silencing by DNA methylation, we performed expression analysis by RT-PCR for eight genes, TFAP2C, NKX2-5, GALR2, DPYS, TFAP2E, CDEP, SPOCK2, and NSE1 selected based on methylation frequency and potential function (Figure 5). Most cell lines chosen for analysis had high methylation level (>50%), and all had very low levels of expression at baseline. Expression of these genes was easily restored after treatment with the demethylating agent, 5-azadC in all cases suggesting silencing related to DNA methylation. The histone deacetylase inhibitor, TSA alone did not restore gene expression at TFAP2C, NKX2-5 and NSE1. The other genes showed increased expression after TSA but to a lesser extent than after 5-azadC treatment. For 5-azadC and TSA combined treatment, TFAP2C, TFAP2E, CDEP, SPOCK2 and NSE1 showed synergistic effects on expression.

### Discussion

To isolate novel methylated genes in cancer, we applied MCA-RDA to prostate cancer cell lines and identified 34 promoter-associated CpG islands differently methylated compared to normal control. All of 17 genes investigated had hypermethylation in at least 2 cell lines of a 21-cell line panel containing prostate cancer, colon cancer, leukemia, and breast cancer. We thus confirmed that MCA-RDA is a very powerful tool to identify sequences methylated at multiple loci. MCA-RDA with repetitive subtractive hybridization is, however, labor intensive and costly, and requires hundreds of clones to be sequenced. A recent improvement was to couple MCA with microarrays [17] thus improving the yield of the method. MCA could also potentially be coupled with deep sequencing technology [18].

In most of the cases, cancer cell lines exhibited higher levels of CpG island hypermethylation than primary cancers as previously shown [19]. For example, most prostate and colon cancer cell lines showed hypermethylation at FOXN4 and GALR2, but primary prostate cancers showed lower frequency methylation compared to primary colon cancer. Therefore, it seems likely that part of the hypermethylation events displayed in cancer cell lines come from repeated passages and adaptation to culture environment. In addition, compared to an immortalized cell line (PWR-1E) that was established in 1996, SVHUC cell line, established in 1987 [20], showed increased methylation of some genes, likely derived from reiterated passages in cell culture. Nevertheless, our results also show that cancer cell lines maintain hypermethylation specificity because most methylation events in cancer cell lines also occur in some primary cancers.

DNA methylation provides a powerful diagnostic biomarker for cancer. It was recently estimated that hypermethylation is one order of magnitude more frequent than mutation in cancer [21] DNA methylation could be useful for disease diagnosis in uncertain cancer, or for screening in body fluids [22]. In this study, we identify high sensitivity markers for prostate cancer, colon cancer and breast cancer, which could be worth testing in appropriate surrogate samples (e.g. ejaculate, urine for prostate cancer; serum, stool for colon cancer; nipple aspirate for breast cancer etc.). It is also interesting that some of the genes show substantial methylation in ‘adjacent’ normal tissues. It remains to be seen whether this is related to age-dependent methylation [23], [24] or to a field defect specific to patients with cancer [25]. It is also interesting to note tissue specific differences in methylation. For example, NKX2-5 is methylated in prostate and colon cancer, but rarely in breast cancer. GALR2 is hypermethylated in colon cancer and breast cancer but rarely in prostate cancer. The mechanisms underlying such differences deserve further investigation.

Our data identified TFAP2C and TFAP2E as potentially important new targets of transcriptional silencing in colon cancer. The AP-2 family of transcription factors consists of five homologous proteins, AP-2*α*, AP-2*β*, AP-2*γ*, AP-2*δ*, and AP-2*ε*. AP-2 transcription factors play important roles in embryonic development by influencing the differentiation, proliferation, and survival of cells. The overexpression of AP-2 in HepG2 human hepatoblastoma and SW480 human colon adenocarcinoma cells inhibited cell division and colony formation [26]. Importantly, re-expression of AP-2 in AP-2 negative melanoma cells suppressed their tumorigenicity and metastatic potential in nude mice [27]. These data suggest that AP-2 transcription factors are involved in the maintenance of a proliferative and undifferentiated state of cells, a characteristic not only important to embryonic development but also to tumorigenesis.

Some of the other genes identified could also be functionally important. However, the methylation observed here may also simply reflect the global redistribution of 5-methylcytosine during cancer development [28]. Thus, these clones should be functionally investigated before conclusions can be made regarding their role in cancer. In conclusion, our data identify potentially important new targets of transcriptional silencing in cancer, and provide new biomarkers that could be useful in screening for prostate cancer, colon cancer and breast cancer.

## Supporting Information

Table S1 Methylation densities (%) of 17 genes investigated in cell lines.
(0.10 MB DOC)

Table S2 Methylation densities (%) in prostate cancers and paired normal prostate samples.
(0.12 MB DOC)

Table S3 Methylation densities (%) in colorectal cancers and paired normal colon samples.
(0.12 MB DOC)

Table S4 Methylation densities (%) in breast cancers and paired normal breast samples.
(0.13 MB DOC)
