# Identification of Novel Tumor Markers in Prostate, Colon and Breast Cancer by Unbiased Methylation Profiling

**DOI:** 10.1371/annotation/2548989f-1f13-4ea5-8af8-62420b0a590e

**Published:** 2008-05-06

**Authors:** Woonbok Chung, Bernard Kwabi-Addo, Michael Ittmann, Jaroslav Jelinek, Lanlan Shen, Yinhua Yu, Jean-Pierre J. Issa

There was an error in the author affiliations. The correct affiliations should appear as shown here:

1 Department of Leukemia, M. D. Anderson Cancer Center, Houston, Texas, United States of America, 2 Department of Pathology, Baylor College of Medicine, Houston, Texas, United States of America, 3 Department of Experimental Therapeutics, M. D. Anderson Cancer Center, Houston, Texas, United States of America

