# Supplementary material for: Identification of Novel Tumor Markers in Prostate, Colon and Breast Cancer by Unbiased Methylation Profiling
Source: PLoS One. 2008 Apr 30;3(4):e2079. doi: 10.1371/journal.pone.0002079 (PMC2323612; doi:10.1371/journal.pone.0002079)
Supplement: Table S1 — Methylation densities (%) of 17 genes investigated in cell lines. (0.10 MB DOC) [file pone.0002079.s001.doc]

**Supplementary Table S1. Methylation densities (%) of 17 genes investigated in cell lines.**

| Cell line | FOXN4 | EKI2 | TFAP2C | IRX5 | DEP | BTBD14A | NSE1 | SLC16A12 | TOX | PAX9 | TFAP2E | GALR2 | NKX2-5 | DPYS | CLSTN1 | EGFR5 | SPOCK2 |
| --- | --- | --- | --- | --- | --- | --- | --- | --- | --- | --- | --- | --- | --- | --- | --- | --- | --- |
| DU145 | 15 | 5 | 1 | 20 | 4 | 7 | 68 | 39 | 94 | 8 | 2 | 54 | 54 | 85 | 9 | 96 | 46 |
| PC3 | 24 | 0 | 1 | 41 | 0 | 14 | 2 | 77 | 3 | 3 | 40 | 44 | 77 | 65 | 6 | 91 | 82 |
| LNCaP | 67 | 18 | 1 | 0 | 44 | 0 | 29 | 80 | 9 | 3 | 66 | 15 | 82 | 45 | 87 | 94 | 75 |
| CaCO2 | 3 | 0 | 1 | 0 | 10 | 0 | 6 | 32 | 8 | 3 | 34 | 71 | 84 | 82 | 23 | 95 | 34 |
| HCT113 | 22 | 0 | 1 | 85 | 0 | 6 | 70 | 97 | 4 | 2 | 2 | 83 | 87 | 93 | 64 | 96 | 87 |
| HT29 | 75 | 78 | 42 | 0 | 0 | 0 | 1 | 95 | 3 | 2 | 3 | 77 | 86 | 86 | 1 | 94 | 79 |
| LOVO | 91 | 0 | 52 | 33 | 0 | 0 | 51 | 85 | 58 | 3 | 2 | 48 | 35 | 84 | 27 | 94 | 82 |
| RKO | 75 | 0 | 6 | 77 | 23 | 0 | 14 | 96 | 64 | 3 | 46 | 79 | 35 | 93 | 90 | 96 | 87 |
| SW48 | 98 | 39 | 43 | 59 | 0 | 0 | 25 | 92 | 96 | 3 | 89 | 87 | 1 | 92 | 42 | 97 | 85 |
| HL60 | 0 | 4 | 87 | 0 | 99 | 0 | 83 | 98 | 3 | 64 | 86 | 80 | 89 | 92 | 93 | 94 | 34 |
| Jurkat | 0 | 38 | 91 | 0 | 30 | 0 | 90 | 97 | 2 | 22 | 86 | 84 | 88 | 94 | 53 | 97 | 25 |
| Raji | 77 | 0 | 87 | 95 | 99 | 87 | 80 | 96 | 4 | 89 | 76 | 79 | 89 | 93 | 97 | 96 | 83 |
| RS4;11 | 50 | 0 | 82 | 0 | 52 | 68 | 86 | 89 | 4 | 98 | 90 | 60 | 79 | 93 | 18 | 3 | 0 |
| K562 | 23 | 33 | 1 | 0 | 20 | 0 | 10 | 32 | 4 | 3 | 43 | 35 | 25 | 62 | 5 | 14 | 71 |
| BT474 | 21 | 0 | 6 | 0 | 0 | 0 | 16 | 2 | 96 | 3 | 8 | 57 | 82 | 85 | 4 | 97 | 74 |
| Cama1 | 8 | 0 | 1 | 0 | 0 | 0 | 9 | 75 | 95 | 2 | 38 | 65 | 82 | 83 | 3 | 95 | 79 |
| HTB126 | 8 | 0 | 23 | 0 | 71 | 0 | 45 | 89 | 2 | 2 | 11 | 9 | 2 | 52 | 5 | 93 | 44 |
| MCF7 | 47 | 0 | 1 | 0 | 0 | 0 | 14 | 91 | 95 | 2 | 3 | 77 | 85 | 66 | 0 | 93 | 80 |
| Hep3B | 9 | 0 | 26 | 0 | 0 | 0 | 7 | 19 | 6 | 3 | 1 | 4 | 0 | 93 | 6 | 94 | 4 |
| HepG2 | 3 | 9 | 35 | 77 | 0 | 0 | 42 | 19 | 21 | 30 | 24 | 2 | 22 | 18 | 7 | 93 | 0 |
| SK-Hep1 | 1 | 9 | 1 | 0 | 14 | 6 | 64 | 8 | 95 | 3 | 17 | 32 | 0 | 59 | 28 | 88 | 71 |
| NL | 0 | 0 | 1 | 0 | 0 | 0 | 5 | 5 | 2 | 5 | 9 | 7 | 2 | 10 | 11 | 9 | 0 |
| SVHUC | 0 | 0 | 1 | 0 | 0 | 2 | 2 | 3 | 3 | 3 | 24 | 30 | 53 | 48 | 70 | 88 | 84 |

NL; Normal Leukocytes
