# Supplementary material for: Identification of Novel Tumor Markers in Prostate, Colon and Breast Cancer by Unbiased Methylation Profiling
Source: PLoS One. 2008 Apr 30;3(4):e2079. doi: 10.1371/journal.pone.0002079 (PMC2323612; doi:10.1371/journal.pone.0002079)
Supplement: Table S2 — Methylation densities (%) in prostate cancers and paired normal prostate samples. (0.12 MB DOC) [file pone.0002079.s002.doc]

**Supplementary Table S2. Methylation densities (%) in prostate cancers and paired normal prostate samples.**

| Prostate Normal vs. Cancer | NKX2-5 | CALSTN1 | SPOCK2 | NSE1 | SLC16A12 | FOXN4 | GALR2 | DPYS |
| --- | --- | --- | --- | --- | --- | --- | --- | --- |
| 1 N | 8 | 5 | 5 | 12 | 4 | 3 | 1 | 19 |
| 2 N | 4 | 1 | 14 | 14 | 21 | 3 | 3 | 39 |
| 3 N | 4 | 2 | 6 | 18 | 12 | 4 | 1 | 33 |
| 4 N | 4 | 3 | 6 | 16 | 5 | 4 | 1 | 20 |
| 5 N | 9 | 8 | 9 | 10 | 15 | 2 | 9 | 31 |
| 6 N | 1 | 0 | 9 | 15 | 4 | 2 | 1 | 29 |
| 7 N | 3 | 0 | 5 | 6 | 6 | 2 | 5 | 12 |
| 8 N | 3 | 3 | 8 | 9 | 7 | 5 | 2 | 23 |
| 9 N | 0 | 0 | 10 | 9 | 8 | 3 | 1 | 22 |
| 10 N | 5 | 3 | 7 | 6 | 5 | 0 | 1 | 12 |
| 11 N | 5 | 1 | 8 | 9 | 5 | 1 | 3 | 23 |
| 12 N | 0 | 1 | 10 | 12 | 7 | 3 | 4 | 24 |
| 13 N | 0 | 3 | 4 | 7 | 5 | 2 | 2 | 18 |
| 14 N | 0 | 5 | 2 | 9 | 7 | 3 | 0 | 22 |
| 15 N | 0 | 0 | 3 | 5 | 4 | 1 | 0 | 9 |
| 16 N | 0 | 3 | 10 | 12 | 24 | 2 | 2 | 36 |
| 17 N | 5 | 6 | 13 | 10 | 20 | 1 | 0 | 39 |
| 18 N | 0 | 1 | 6 | 6 | 5 | 3 | 1 | 22 |
| 19 N | 0 | 3 | 7 | 10 | 7 | 3 | 1 | 27 |
| 20 N | 0 | 2 | 9 | 3 | 6 | 3 | 2 | 18 |
| 1 T | 40 | 28 | 30 | 31 | 31 | 6 | 7 | 66 |
| 2 T | 17 | 5 | 19 | 16 | 22 | 5 | 4 | 49 |
| 3 T | 7 | 10 | 20 | 31 | 28 | 6 | 7 | 54 |
| 4 T | 7 | 17 | 10 | 28 | 15 | 2 | 6 | 45 |
| 5 T | 40 | 43 | 25 | 31 | 45 | 12 | 4 | 66 |
| 6 T | 26 | 26 | 28 | 26 | 31 | 13 | 4 | 57 |
| 7 T | 5 | 5 | 3 | 21 | 12 | 4 | 3 | 26 |
| 8 T | 15 | 13 | 9 | 16 | 16 | 7 | 2 | 37 |
| 9 T | 10 | 10 | 12 | 13 | 19 | 5 | 1 | 43 |
| 10 T | 30 | 7 | 15 | 16 | 16 | 3 | 3 | 35 |
| 11 T | 10 | 11 | 18 | 28 | 15 | 3 | 7 | 41 |
| 12 T | 1 | 24 | 22 | 11 | 27 | 3 | 4 | 42 |
| 13 T | 0 | 6 | 17 | 26 | 23 | 3 | 0 | 40 |
| 14 T | 1 | 8 | 4 | 15 | 7 | 2 | 1 | 27 |
| 15 T | 0 | 6 | 4 | 25 | 17 | 1 | 2 | 61 |
| 16 T | 0 | 2 | 11 | 15 | 11 | 1 | 8 | 14 |
| 17 T | 29 | 5 | 17 | 21 | 33 | 5 | 7 | 47 |
| 18 T | 1 | 3 | 6 | 19 | 25 | 1 | 10 | 50 |
| 19 T | 41 | 11 | 26 | 19 | 40 | 3 | 4 | 37 |
| 20 T | 28 | 19 | 27 | 29 | 37 | 3 | 3 | 44 |
| Mean of Normal (n=20) | 2.7 | 2.5 | 7.5 | 10.3 | 9 | 2.4 | 2 | 24.2 |
| Mean of Tumor (n=20) | 14 | 12.1 | 15.2 | 20.5 | 22 | 4.3 | 4.3 | 42.7 |
| Normal Mean+2SD | 8.6 | 7.1 | 13.9 | 17.5 | 21.6 | 4.8 | 6.3 | 41.7 |
