# Supplementary material for: Identification of Novel Tumor Markers in Prostate, Colon and Breast Cancer by Unbiased Methylation Profiling
Source: PLoS One. 2008 Apr 30;3(4):e2079. doi: 10.1371/journal.pone.0002079 (PMC2323612; doi:10.1371/journal.pone.0002079)
Supplement: Table S3 — Methylation densities (%) in colorectal cancers and paired normal colon samples. (0.12 MB DOC) [file pone.0002079.s003.doc]

**Supplementary Table S3. Methylation densities (%) in colorectal cancers and paired normal colon samples.**

| Colorectal Normal vs. Cancer | NKX2-5 | SPOCK2 | GALR2 | FOXN4 | TPAP2C | TFAP2E | IRX-5 | DPYS | SLC16A12 |
| --- | --- | --- | --- | --- | --- | --- | --- | --- | --- |
| 1 N | 4 | 8 | 6 | 0 | 5 | 14 | 16 | 23 | 6 |
| 2 N | 2 | 12 | 7 | 0 | 3 | 8 | 8 | 17 | 7 |
| 3 N | 4 | 4 | 8 | 0 | 3 | 8 | 15 | 24 | 5 |
| 4 N | 2 | 6 | 9 | 0 | 4 | 9 | 15 | 22 | 6 |
| 5 N | 8 | 16 | 16 | 0 | 3 | 6 | 7 | 27 | 13 |
| 6 N | 2 | 0 | 3 | 0 | 4 | 8 | 4 | 12 | 6 |
| 7 N | 6 | 15 | 11 | 0 | 4 | 12 | 16 | 29 | 8 |
| 8 N | 7 | 10 | 15 | 0 | 6 | 14 | 23 | 29 | 12 |
| 9 N | 2 | 8 | 6 | 0 | 5 | 12 | 16 | 19 | 7 |
| 10 N | 2 | 10 | 14 | 0 | 5 | 9 | 9 | 26 | 9 |
| 11 N | 3 | 5 | 8 | 0 | 2 | 15 | 9 | 21 | 8 |
| 12 N | 6 | 6 | 9 | 0 | 4 | 15 | 21 | 20 | 13 |
| 13 N | 8 | 3 | 6 | 0 | 2 | 8 | 26 | 25 | 9 |
| 14 N | 3 | 4 | 5 | 0 | 2 | 6 | 16 | 13 | 9 |
| 15 N | 11 | 5 | 9 | 0 | 3 | 6 | 11 | 20 | 7 |
| 16 N | 8 | 1 | 11 | 0 | 4 | 12 | 16 | 16 | 11 |
| 17 N | 2 | 4 | 6 | 0 | 2 | 14 | 6 | 22 | 9 |
| 18 N | 12 | 11 | 5 | 0 | 1 | 7 | 11 | 15 | 8 |
| 19 N | 12 | 4 | 6 | 0 | 2 | 7 | 3 | 21 | 16 |
| 20 N | 14 | 4 | 7 | 0 | 1 | 6 | 8 | 12 | 6 |
| 1 T | 36 | 41 | 43 | 0 | 3 | 9 | 9 | 41 | 51 |
| 2 T | 25 | 36 | 30 | 0 | 3 | 11 | 0 | 31 | 26 |
| 3 T | 0 | 24 | 20 | 0 | 3 | 4 | 3 | 40 | 4 |
| 4 T | 45 | 36 | 53 | 55 | 40 | 30 | 8 | 47 | 50 |
| 5 T | 50 | 43 | 60 | 26 | 16 | 11 | 0 | 51 | 61 |
| 6 T | 14 | 0 | 27 | 10 | 26 | 6 | 0 | 36 | 52 |
| 7 T | 2 | 18 | 19 | 0 | 10 | 16 | 13 | 42 | 42 |
| 8 T | 28 | 26 | 53 | 25 | 3 | 11 | 0 | 43 | 48 |
| 9 T | 0 | 25 | 32 | 0 | 3 | 14 | 6 | 34 | 37 |
| 10 T | 0 | 0 | 42 | 5 | 14 | 10 | 12 | 34 | 32 |
| 11 T | 33 | 10 | 39 | 0 | 2 | 12 | 2 | 43 | 15 |
| 12 T | 54 | 21 | 45 | 51 | 2 | 8 | 6 | 37 | 55 |
| 13 T | 2 | 0 | 11 | 0 | 3 | 9 | 7 | 22 | 8 |
| 14 T | 0 | 14 | 12 | 0 | 2 | 6 | 0 | 16 | 20 |
| 15 T | 39 | 16 | 21 | 0 | 1 | 4 | 10 | 31 | 25 |
| 16 T | 21 | 0 | 23 | 0 | 5 | 11 | 8 | 25 | 30 |
| 17 T | 59 | 20 | 50 | 57 | 61 | 10 | 2 | 64 | 60 |
| 18 T | 23 | 12 | 38 | 20 | 2 | 4 | 10 | 32 | 24 |
| 19 T | 36 | 27 | 17 | 0 | 4 | 6 | 12 | 49 | 61 |
| 20 T | 23 | 6 | 13 | 0 | 3 | 7 | 8 | 21 | 10 |
| Mean of Normal (n=20) | 5.9 | 6.8 | 8.3 | 0 | 3 | 9.9 | 12.8 | 20.5 | 8.5 |
| Mean of Tumor (n=20) | 24.4 | 18.7 | 32.5 | 12.4 | 10.1 | 9.9 | 5.7 | 37 | 35.3 |
| Normal Mean+2SD | 14 | 15.4 | 15.4 | 0 | 5.9 | 16.4 | 25.4 | 31.1 | 14.3 |
