# Supplementary material for: Identification of Novel Tumor Markers in Prostate, Colon and Breast Cancer by Unbiased Methylation Profiling
Source: PLoS One. 2008 Apr 30;3(4):e2079. doi: 10.1371/journal.pone.0002079 (PMC2323612; doi:10.1371/journal.pone.0002079)
Supplement: Table S4 — Methylation densities (%) in breast cancers and paired normal breast samples. (0.13 MB DOC) [file pone.0002079.s004.doc]

**Supplementary Table S4. Methylation densities (%) in breast cancers and paired normal breast samples.**

| Breast  Normal vs. Cancer | SLC16A12 | DPYS | NKX2-5 | GALR2 | SPOCK2 | TOX | EGFR5 |
| --- | --- | --- | --- | --- | --- | --- | --- |
| 1N | 7 | 4 | 2 | 1 | 3 | 4 | 30 |
| 2N | 6 | 5 | 3 | 2 | 2 | 3 | 11 |
| 3N | 7 | 5 | 2 | 1 | 4 | 3 | 35 |
| 4N | 8 | 6 | 2 | 2 | 3 | 3 | 25 |
| 5N | 5 | 4 | 2 | 1 | 3 | 3 | 19 |
| 6N | 3 | 3 | 3 | 1 | 2 | 3 | 9 |
| 7N | 4 | 4 | 2 | 1 | 3 | 4 | 13 |
| 8N | 4 | 6 | 3 | 1 | 1 | 2 | 6 |
| 9N | 5 | 3 | 4 | 2 | 2 | 7 | 11 |
| 10N | 5 | 5 | 2 | 2 | 3 | 3 | 33 |
| 11N | 4 | 4 | 2 | 2 | 2 | 2 | 12 |
| 12N | 4 | 4 | 3 | 1 | 3 | 3 | 3 |
| 13N | 3 | 4 | 2 | 2 | 2 | 3 | 5 |
| 14N | 4 | 4 | 3 | 1 | 2 | 2 | 7 |
| 15N | 3 | 2 | 2 | 1 | 2 | 3 | 4 |
| 16N | 4 | 5 | 2 | 2 | 3 | 2 | 23 |
| 17N | 4 | 13 | 2 | 1 | 4 | 26 | 33 |
| 18N | 3 | 8 | 3 | 5 | 2 | 8 | 4 |
| 19N | 5 | 5 | 1 | 1 | 1 | 2 | 53 |
| 20N | 4 | 3 | 3 | 2 | 2 | 3 | 6 |
| 21N | 7 | 5 | 3 | 2 | 3 | 7 | 14 |
| 22N | 4 | 4 | 2 | 1 | 2 | 3 | 6 |
| 23N | 3 | 4 | 1 | 1 | 3 | 3 | 28 |
| 24N | 10 | 6 | 2 | 6 | 5 | 6 | 27 |
| 1T | 30 | 12 | 2 | 12 | 22 | 26 | 38 |
| 2T | 4 | 31 | 2 | 1 | 6 | 3 | 61 |
| 3T | 3 | 15 | 3 | 7 | 18 | 10 | 54 |
| 4T | 2 | 39 | 1 | 62 | 1 | 4 | 70 |
| 5T | 43 | 28 | 2 | 13 | 31 | 2 | 47 |
| 6T | 6 | 7 | 4 | 4 | 3 | 2 | 37 |
| 7T | 14 | 23 | 3 | 7 | 6 | 22 | 19 |
| 8T | 51 | 40 | 1 | 45 | 9 | 2 | 52 |
| 9T | 5 | 6 | 3 | 2 | 3 | 8 | 34 |
| 10T | 14 | 8 | 2 | 4 | 2 | 6 | 15 |
| 11T | 4 | 10 | 1 | 1 | 5 | 2 | 42 |
| 12T | 54 | 14 | 3 | 4 | 30 | 39 | 48 |
| 13T | 5 | 19 | 1 | 8 | 1 | 3 | 91 |
| 14T | 4 | 6 | 2 | 0 | 1 | 2 | 63 |
| 15T | 8 | 19 | 20 | 16 | 46 | 34 | 60 |
| 16T | 4 | 45 | 2 | 4 | 1 | 2 | 42 |
| 17T | 3 | 36 | 2 | 3 | 23 | 54 | 71 |
| 18T | 14 | 61 | 2 | 59 | 2 | 64 | 47 |
| 19T | 2 | 55 | 5 | 10 | 6 | 59 | 76 |
| 20T | 6 | 6 | 2 | 5 | 9 | 57 | 64 |
| 21T | 50 | 12 | 5 | 16 | 48 | 78 | 77 |
| 22T | 6 | 24 | 2 | 3 | 2 | 2 | 45 |
| 23T | 5 | 17 | 2 | 7 | 15 | 3 | 49 |
| 24T | 6 | 18 | 7 | 17 | 22 | 30 | 33 |
| Mean of Normal (n=24) | 4.8 | 4.7 | 2.3 | 1.8 | 2.5 | 4.6 | 17.4 |
| Mean of Tumor (n=24) | 14.3 | 22.9 | 3.2 | 12.9 | 13.1 | 21.5 | 51.4 |
| Normal mean + 2SD | 8.5 | 8.8 | 3.7 | 4.3 | 4.1 | 14.3 | 43.7 |
